# Predicting immune-related adverse events in patients with melanoma: the role of interleukin-7 rs16906115 polymorphism and lymphocyte dynamics

**DOI:** 10.3389/fimmu.2025.1616325

**Published:** 2025-06-26

**Authors:** Fatma Pınar Açar, Caner Acar, Damla Gunenc, Çağlar Arisoy, Asli Ece Solmaz, Asli Gecgel, Haydar Çağatay Yüksel, Gökhan Şahin, Oguzcan Ozkan, Zeynep Sila Gokdere, Nilay Duman, Burçak Karaca

**Affiliations:** ^1^ Division of Medical Oncology, Department of Internal Medicine, Ege University Medical Faculty, Izmir, Türkiye; ^2^ Division of Medical Oncology, Hatay Education and Research Hospital, Hatay, Türkiye; ^3^ Department of Medical Genetics, Ege University Medical Faculty, Izmir, Türkiye; ^4^ Department of Dermatology, Ege University Medical Faculty, Izmir, Türkiye

**Keywords:** immune checkpoint inhibitors, rs16906115 polymorphism, lymphocyte stability index, melanoma, genetic predisposition, immune-related adverse events, cancer immunotherapy

## Abstract

**Introduction:**

Immune checkpoint inhibitors (ICIs) have transformed the therapeutic landscape of malignant melanoma; however, they are frequently associated with immune-related adverse events (irAEs). Emerging evidence suggests that genetic predispositions, including interleukin-7 (IL-7) gene variants, may influence the risk of these toxicities.

**Methods:**

In this single-center retrospective study, we investigated the potential utility of IL-7 rs16906115 polymorphism and lymphocyte stability index (LSI) in predicting susceptibility to irAEs among 96 melanoma patients treated with ICIs.

**Results:**

Genotyping revealed a minor allele frequency of 8.3% for rs16906115. Logistic regression analysis indicated that carriers of the minor allele had a significantly increased risk of all-grade irAEs compared to reference allele carriers (adjusted OR: 3.93; 95%CI:1.13–13.64; p=0.031). Subgroup analyses revealed a significant increase in risk across endocrine, non-cutaneous, multiple, low-grade, and early onset (<3 months) irAEs. While neither baseline lymphocyte count nor LSI predicted overall irAE incidence, an elevated LSI emerged as a key risk factor for early steroid-requiring irAEs (adjusted OR:3.79; 95% CI: 1.14–12.61; p =0.030).

**Discussion:**

These findings from a Turkish cohort corroborate earlier European studies suggesting that rs16906115 minor allele carriage may be a genetic risk factor for irAEs. Furthermore, LSI may serve as a dynamic biomarker for predicting early steroid-requiring irAEs. Prospective multicenter studies among diverse populations are warranted to validate these findings.

## 1 Introduction

Immune checkpoint inhibitors (ICIs) that target cytotoxic T-lymphocyte antigen-4 (CTLA-4) and programmed cell death protein-1 (PD-1) have revolutionized the treatment of malignant melanoma (1). Moreover, due to their demonstrated efficacy in the metastatic setting, ICIs have subsequently been employed in the adjuvant and neoadjuvant settings, becoming a mainstay in the management of this disease (2). ICIs enhance T-cell activation and stimulate robust anti-tumour response by eliminating immunosuppressive signals in the tumour microenvironment (3).

However, unregulated immune activation may lead to immune-related adverse events (irAEs). The underlying mechanisms of irAE development include the production of antibodies by autoreactive B-cells, cytotoxic T-cell infiltration, and immune responses mediated by inflammatory cytokines, although its exact cause and the contributing factors remain unclear (4). The skin, colon, endocrine organs, and liver are the sites most commonly affected by irAEs, while infrequent and potentially fatal irAEs have also been reported (5, 6). In some studies, the incidence of high-grade (grade ≥ 3) irAEs reached approximately 20% in patients receiving anti-PD-1 therapy and approximately 59% in those receiving combined anti-CTLA-4/PD-1 therapy (1, 7).

The clinical course of irAEs exhibits significant variability, ranging from multi-organ involvement to minimal or no toxicity. Some patients may experience life-threatening irAEs, even necessitating the discontinuation of ICI therapy. In addition, the timing of irAE onset differs markedly among patients. Some may present with irAEs after the first cycle of therapy, whereas others may develop late-onset irAEs, sometimes occurring after treatment discontinuation (8, 9). There is also ongoing debate regarding whether the immunosuppressive treatments used to manage irAEs undermine the efficacy of ICIs, leading to further uncertainty (10).

These challenges highlight the urgent need to identify predictive factors concerning irAE development. Thus, numerous studies have investigated potential biomarkers—including blood-based markers, gut microbiome profiles, and autoantibodies—although no reliable predictor has yet been introduced into routine clinical practice (11). It has been proposed that genetic factors may confer susceptibility to irAEs in patients receiving ICIs. Multiple recent studies have reported associations between germline genetic variations and the risk of irAE development (12). A recent genome-wide association study identified an association between the minor allele of the single-nucleotide polymorphism (SNP) rs16906115 in the interleukin-7 (IL-7) gene, lymphocyte stability, and all-grade irAEs (13). Subsequent replication in a cohort of patients with melanoma supported these findings, suggesting that increased IL-7 expression in B-cells might be the principal mechanism involved (14). IL-7 is a key regulator of the adaptive immune system: besides its role in B-cell development, it supports the proliferation and homeostasis of naive and memory T-cells (15).

Following these studies, a study in the Japanese population failed to validate the association between IL-7 gene polymorphism and an increased risk of irAEs, underscoring the necessity of confirming such genetic associations in diverse ethnic groups (16). Therefore, our study aimed to evaluate the frequency of the IL-7 rs16906115 gene polymorphism and its potential association with irAE development among melanoma patients in the Turkish population receiving ICI therapy. We further explored the effects of the rs16906115 polymorphism on specific irAE subgroups and assessed whether the lymphocyte stability index (LSI) may also serve as a risk factor.

## 2 Materials and methods

This single‐center retrospective study was conducted at Ege University’s Medical Oncology Clinic. Patients diagnosed with malignant melanoma who were treated with anti‐PD‐1 ± CTLA‐4 therapy between January 2016 and September 2024 were included. This study adhered to the Good Clinical Practice guidelines and complied with the ethical principles of the Declaration of Helsinki. Ethical approval was obtained from the Institutional Ethical Review Board of Ege University Hospital (Approval no. 23‐12.1T/58). All of the participating patients provided written informed consent.

Following enrolment, 20 mL of peripheral blood was collected from each patient for analysis. Genomic DNA was extracted from whole blood samples using the GeneAll Exgene Blood SV Mini Kit (GeneAll Biotechnology, South Korea) according to the manufacturer’s protocol. The concentration and purity of the extracted DNA were assessed using a spectrophotometer, and the samples were stored at -20°C until further analysis. Polymerase chain reaction (PCR) was performed to detect the rs16906115 C/T variants using a Bio-Rad thermal cycler (Bio-Rad Laboratories, USA). The genotyping assay included rs16906115 C master mix, rs16906115 T master mix (SNP Biotechnology, Turkey), and rs16906115 C/T heterozygous control DNA. Each reaction consisted of 20 μL of master mix and 5 μL of extracted DNA, making a final reaction volume of 25 μL per well. The PCR amplification was carried out under the following thermal cycling conditions: an initial denaturation at 95°C for 3 minutes, followed by 30 cycles of denaturation at 95°C for 15 seconds and annealing/extension at 60°C for 1 minute. PCR results were analyzed using the Bio-Rad detection system. Both amplification and melt peak graphs were evaluated to determine the presence of rs16906115 C/T variants. The presence of distinct melt peaks corresponding to the specific genotypes was used for allele discrimination. All experiments were conducted in compliance with relevant laboratory guidelines and quality control measures to ensure the reliability of the results. The minor allele frequency (MAF) of the rs16906115 C/T polymorphism was calculated by dividing the number of copies of the T allele (considered as the minor allele in this case) by the total number of alleles (both T and C alleles) present in the population.

Demographic and clinical parameters, including age, sex, histological subtype, BRAF mutation status, metastatic sites, and the type of ICI used, were recorded. Baseline (pre-treatment) and post-treatment laboratory data, including the lymphocyte count (collected at least 21 days after therapy initiation), lactate dehydrogenase (LDH), C‐reactive protein (CRP), and albumin levels, were noted. The LSI was calculated as the ratio of the post-treatment to the pre-treatment lymphocyte count.

As adjuvant treatment, the patients received 200 mg of pembrolizumab intravenously (IV) every three weeks (Q3W) or nivolumab, administered as either 3 mg/kg IV every two weeks (Q2W) or 240 mg IV Q2W, in accordance with the approved dosing protocol at the time of the treatment initiation. In the metastatic setting, the patients were treated either with anti‐PD‐1 monotherapy (3 mg/kg of nivolumab IV Q2W or 240 mg IV Q2W) or with nivolumab–ipilimumab combination (3 mg/kg of ipilimumab and 1 mg/kg of nivolumab IV Q3W for four cycles), followed by maintenance nivolumab (3 mg/kg or 240 mg IV Q2W). Adverse events (AEs) were graded according to the Common Terminology Criteria for Adverse Events (CTCAE) version 5.0. Here, irAEs and the respective grades were identified from the patients’ electronic medical records and retrospective patient interviews. In the no-irAE (control) group, patients who had received at least two treatment cycles and were followed for at least three months were included.

Comparisons of the categorical variables between groups were performed using the chi‐square test or Fisher’s exact test, depending on the sample size. For the continuous variables, the Mann–Whitney U test was employed. The within‐group changes in the lymphocyte counts were assessed using the Wilcoxon signed‐rank test. Logistic regression analysis was conducted to identify risk factors for developing irAEs. To determine the optimal cut‐off values for the pre-treatment lymphocyte count and the LSI in predicting irAEs, a receiver operating characteristic (ROC) curve analysis was performed using the pROC package in R (version 4.4.2, accessed 25 January 2025). Youden’s index was then used to select the best threshold. Forest plots and box plots were generated in R using the forestplot and ggplot2 packages, respectively. All of the other statistical analyses were performed using jamovi (version 2.3.28; the jamovi project, 2023; https://www.jamovi.org). A p‐value < 0.05 was considered to be statistically significant.

## 3 Results

### 3.1 Patients’ demographic and clinical characteristics

A total of 96 patients who met the inclusion criteria were enrolled in this study. Demographic and clinical characteristics of the patients are shown in **Table 1**. The median (interquartile range [IQR]) age was 54.0 (45.0–63.2) years, and the majority (63.5%) of patients were male. Overall, 54 (56.2%) patients received anti‐PD‐1 therapy, whereas 42 (43.8%) were treated with nivolumab–ipilimumab combination. 62 (64.6%) patients received ICI treatment in the metastatic setting and 34 (35.4%) in the adjuvant setting.

**Table 1:** Demographic and clinical characteristics of study cohort.

Variable	Category	CC (n=80)	CT (n= 16)	Total (n=96)	p
Age, years	Median (IQR)	54.0 (44.8 to 63.0)	53.5 (45.8 to 67.0)	54.0 (45.0 to 63.2)	0.764
Gender	Male	50 (62.5)	11 (68.8)	61 (63.5)	0.850
	Female	30 (37.5)	5 (31.2)	35 (36.5)	
Histological subgroup	SSM	22 (27.5)	1 (6.2)	23 (24.0)	0.249
	NM	13 (16.2)	5 (31.2)	18 (18.8)	
	AM	6 (7.5)	1 (6.2)	7 (7.3)	
	NOS	35 (43.8)	9 (56.2)	44 (45.8)	
	UM	4 (5.0)	0 (0.0)	4 (4.2)	
BRAF status	Wild	57 (71.2)	6 (37.5)	63 (65.6)	0.021
	Mutant	23 (28.8)	10 (62.5)	33 (34.4)	
Brain metastasis	No	73 (91.2)	14 (87.5)	87 (90.6)	1.000
	Yes	7 (8.8)	2 (12.5)	9 (9.4)	
Liver metastasis	No	69 (86.2)	14 (87.5)	83 (86.5)	1.000
	Yes	11 (13.8)	2 (12.5)	13 (13.5)	
Bone metastasis	No	63 (78.8)	13 (81.2)	76 (79.2)	1.000
	Yes	17 (21.2)	3 (18.8)	20 (20.8)	
Lung metastasis	No	55 (68.8)	12 (75.0)	67 (69.8)	0.842
	Yes	25 (31.2)	4 (25.0)	29 (30.2)	
ICI type	Anti PD-1	46 (57.5)	8 (50.0)	54 (56.2)	0.783
	Nivo-ipi	34 (42.5)	8 (50.0)	42 (43.8)	
ICI Treatment setting	Adjuvant	29 (36.2)	5 (31.2)	34 (35.4)	0.924
	Metastatic	51 (63.8)	11 (68.8)	62 (64.6)	
ICI treatment duration	Median (IQR)	11.8 (5.3 to 16.7)	11.7 (5.6 to 23.9)	11.8 (5.5 to 17.9)	0.658
Lymphocyte count/μl	Median (IQR)	1980 (1500 to 2470)	1705 (1350 to 2035)	1910 (1470 to 2430)	0.119
LDH	Normal	56 (76.7)	12 (75.0)	68 (76.4)	1.000
	>ULN	17 (23.3)	4 (25.0)	21 (23.6)	

Data are presented as n (%) for categorical variables and median (IQR) for continuous variables. SSM, superficial spreading melanoma; AM, acral melanoma; NM, nodular melanoma; UM, uveal melanoma; NOS, not otherwise specified; LDH, lactate dehydrogenase; ULN, upper limit of normal; ICI, immune checkpoint inhibitor; IQR, interquartile range; Nivo-ipi, nivolumab + ipilimumab combination.

Genotyping analysis of IL‐7 rs16906115 identified 16 (16.7%) patients with the CT genotype and 80 (83.3%) with the CC genotype, while no patients had the TT genotype. The MAF was 8.3%. A comparison of the patients’ characteristics by genotype is presented in **Table 1**. Notably, the CT genotype group had a significantly higher frequency of BRAF mutations when compared to the CC genotype group (62.5% vs. 28.8%, p = 0.021). No other significant difference was observed between the two groups (p > 0.05).

### 3.2 IrAEs

After a median treatment duration of 11.8 months, 47 patients (49%) had developed any irAE, with a median (IQR) time to the irAE of 3.2 (1.3–5.3) months. High‐grade (grade 3–4) irAEs were noted in 21 (21.9%) patients and multiple irAEs occurred in 12 (12.5%). Regardless of grade, irAEs occurred in 59.5% of patients receiving combination ICI therapy and in 40.7% of those receiving anti‐PD‐1 monotherapy. The distribution of irAEs both for the total cohort and by genotype is shown in **Table 2**. Cutaneous irAEs and colitis (each 14.6%) were the most frequent subtypes identified.

**Table 2:** Distribution of immune-related adverse event subtypes by rs16906115 genotype.

IrAE subtypes	Total patient (n=96)		rs16906115 CC (n=80)		rs16906115 CT (n=16)	
	Grade 1-2	Grade 3-4	Grade 1-2	Grade 3-4	Grade 1-2	Grade 3-4
Colitis	7 (7.3)	7 (7.3)	5 (6.3)	5 (6.3)	2 (12.5)	2 (12.5)
Hepatitis	4 (4.2)	8 (8.3)	3 (3.8)	6 (7.5)	1 (6.3)	2 (12.5)
Pneumonitis	6 (6.3)	2 (2.1)	4 (5)	2 (2.5)	2 (1.3)	0 (0)
Endocrine	11 (12.5)	0 (0)	6 (7.5)	0 (0)	5 (31.3)	0 (0)
Cutenous	10 (10.4)	4 (4.2)	7 (8.8)	3 (3.8)	3 (18.8)	1 (6.3)
Artritis	1 (1)	1 (1)	0 (0)	1 (1.3)	1 (6.3)	0 (0)
Pancreatitis	0 (0)	1 (1)	0 (0)	1 (1.3)	0 (0)	0 (0)

Data are presented as n (%).

### 3.3 Lymphocyte dynamics

**Figure 1:**
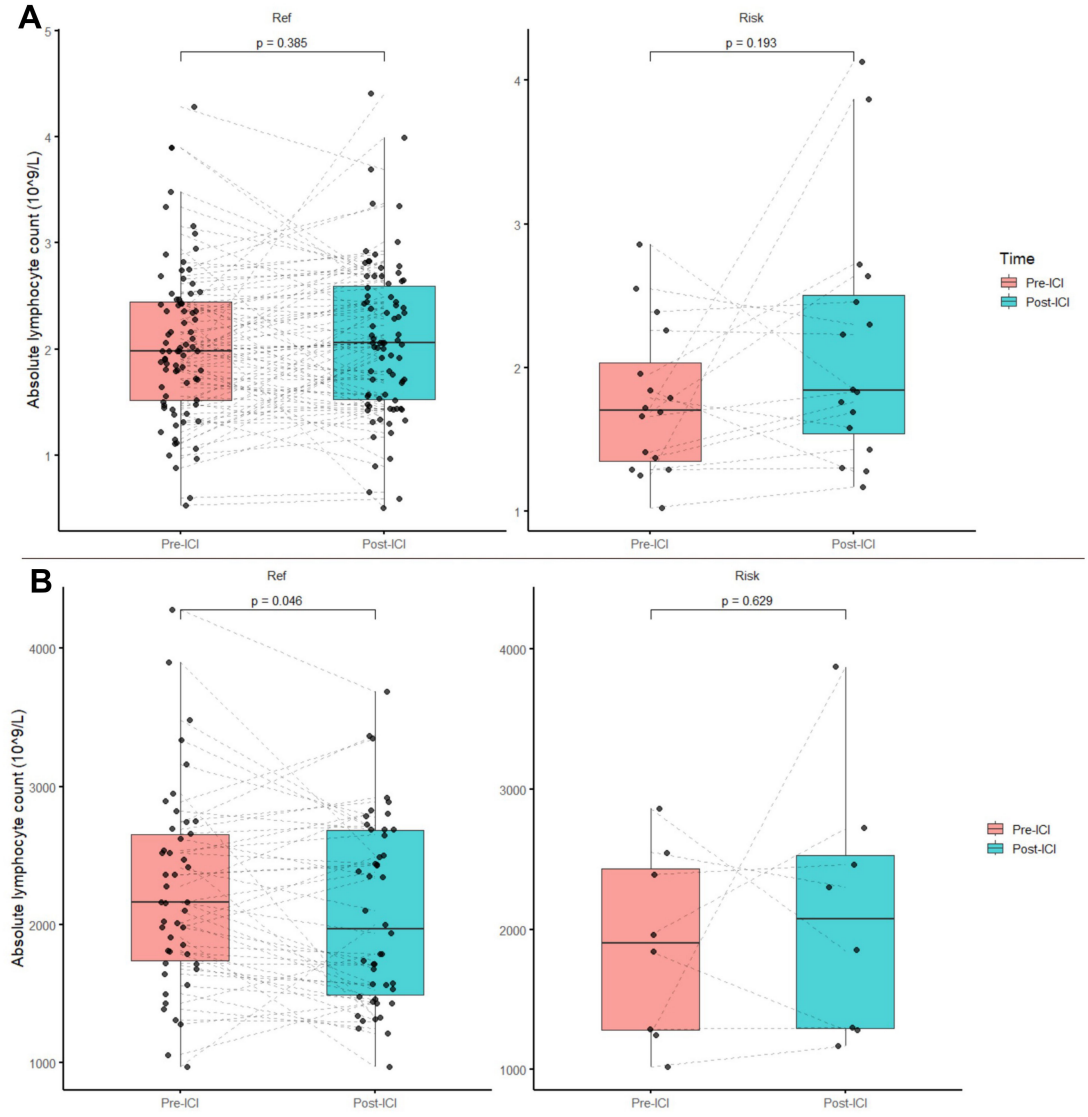
Absolute lymphocyte count changes before and after ICI treatment. **(A)** Total patient population and **(B)** patients receiving Anti-PD-1 therapy.

To evaluate the impact of the SNP rs16906115 on lymphocyte homeostasis, we assessed the pre‐ICI and post‐ICI lymphocyte counts in all of the treated patients. No significant difference in the lymphocyte count changes was observed between the CT and CC genotypes (**Figure 1A**). However, in a subgroup analysis of patients receiving anti‐PD‐1 therapy, the CC genotype group showed a significant decrease in the lymphocyte count after ICI treatment, whereas the patients with the CT genotype maintained lymphocyte stability (**Figure 1B**).

### 3.4 Logistic regression analysis of irAEs

**Figure 2:**
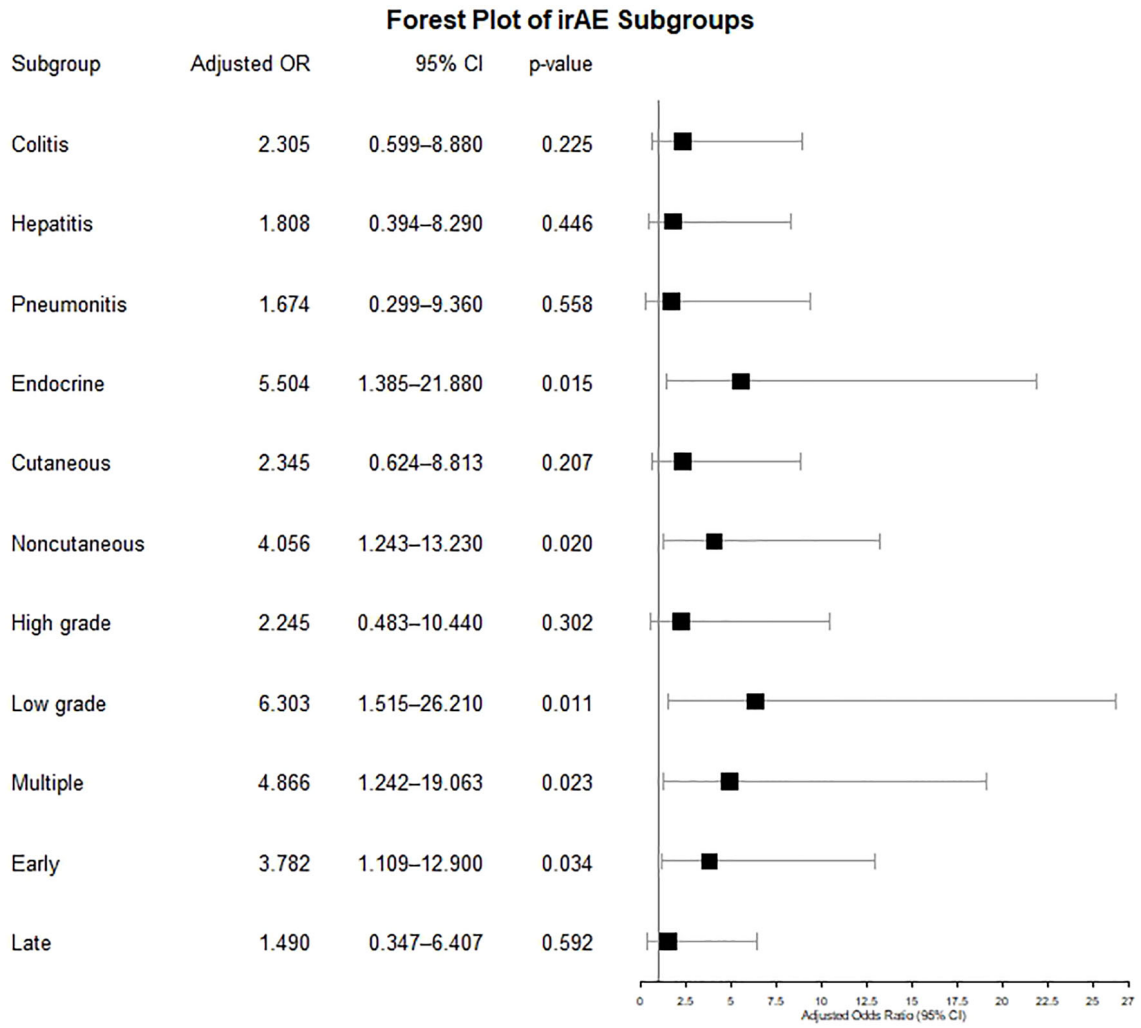
Forest plot showing the risk of IrAE subgroups in minor allele carriers.

The results of the logistic regression analysis of factors predictive of irAEs are shown in **Table 3**. In the univariate analysis, patients with the CT genotype demonstrated a significantly increased risk of irAEs when compared with the CC genotype (p = 0.029). This association remained significant after adjusting for age, sex, and ICI type (adjusted odds ratio [adj OR] 3.925, 95% confidence interval [CI]: 1.130–13.640, p = 0.031). Moreover, in the anti-PD-1 subgroup, the adj OR was 2.843 (95% CI: 0.603–13.42, p = 0.187), whereas in the combination treatment subgroup, it was 6.22 (95% CI: 0.689–56.20, p = 0.104). Further subgroup analyses (**Figure 2**) indicated that the CT carriers had a significantly higher risk of endocrine, non‐cutaneous, low‐grade, multiple, and early (< 3 months) irAEs, while a trend towards an increased risk was observed for the other subgroups. Apart from the genotype, an increase in irAE risk was observed in patients treated with the nivolumab–ipilimumab combination, but it did not reach statistical significance (OR 2.139, 95% CI: 0.941–4.86, p = 0.070). The baseline lymphocyte counts and the LSI did not confer a significant risk of developing any irAE (p = 0.325 and p = 0.155, respectively).

**Table 3:** Logistic regression analysis of factors associated with IrAE.

Variable	OR (95% CI)	p
Age, years	0.983 (0.954-1.010)	0.269
Gender (male vs female)	1.227 (0.533-2.822)	0.630
Histological subtype (acral vs nonacral)	7.024 (0.813-60.72)	0.076
BRAF status (mutant vs wild)	1.169 (0.503-2.724)	0.717
Brain metastasis (yes vs no)	1.339 (0.337-5.331)	0.678
Liver metastasis (no vs yes)	1.639 (0.495-5.430)	0.419
Bone metastasis (yes vs no)	1.054 (0.394-2.823)	0.917
ICI type (nivo-ipi vs anti pd-1)	2.139 (0.941-4.86)	0.070
LDH (>ULN vs normal)	1.04 (0.389-2.762)	0.942
Albumin (low vs high)	1.637 (0.613-4.371)	0.325
CRP (high vs low)	1.307 (0.473-3.612)	0.606
Lymphocyte count	1.000 (0.999-1.00)	0.325
Lymphocyte stability index	2.152 (0.749-6.18)	0.155
IL-7 rs16906115 (CT vs CC)	3.857 (1.145-13.00)	0.029

OR, odds ratio; CI, confidence interval; irAE, immune-related adverse effect; ICI, immune checkpoint inhibitor; LDH, lactate dehydrogenase; ULN, upper limit of normal; CRP, C-reactive protein.

### 3.5 Predictors of steroid‐requiring and early (months) steroid‐requiring irAEs

**Figure 3:**
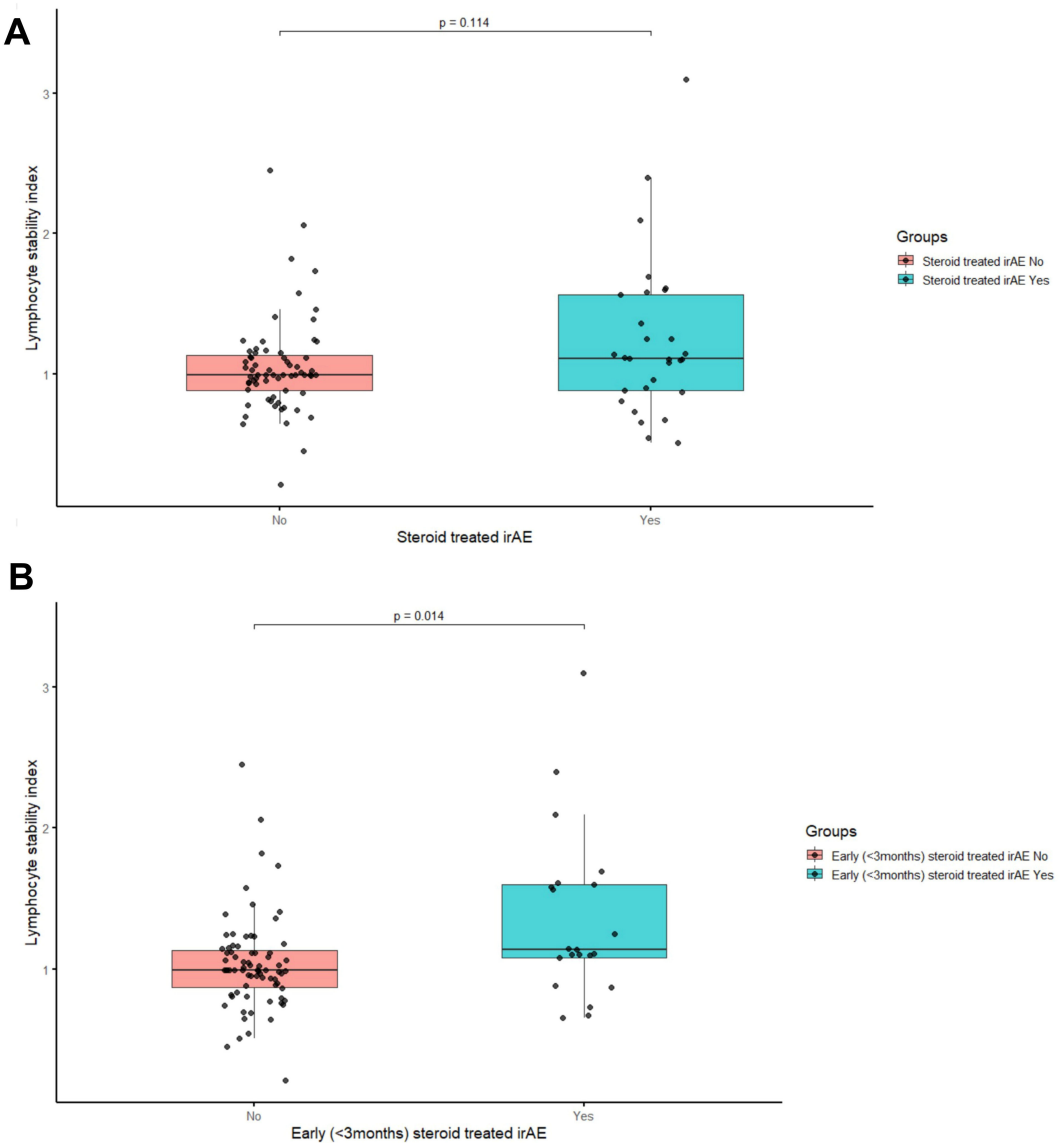
Comparison of lymphocyte stability index. **(A)** Patients With and Without Steroid-Treated IrAE and **(B)** Patients With and Without Early (<3 Months) Steroid-Treated IrAE.

When assessing the LSI, we observed a significant difference in the median LSI between those patients who developed early steroid‐treated irAEs and those who did not (p = 0.014; **Figure 3**). ROC analysis of the LSI revealed an area under the curve of 0.677 (95% CI: 0.530–0.824; **Supplementary Figure S1**). Youden’s index identified 1.07 as the best threshold (76.2% sensitivity, 65.3% specificity). Patients were then stratified into the ‘LSI high’ and ‘LSI low’ groups accordingly. Notably, the frequency of the LSI high classification did not differ between the CT and CC genotypes (p = 0.463).

In the logistic regression analysis of steroid‐requiring irAEs (**Supplementary Table S1**), the adj OR in the LSI high group was 2.875 (95% CI: 1.082–7.640, p = 0.034), while it was 2.711 (95% CI: 0.869–8.462, p = 0.086) for the CT genotype. In terms of the early steroid‐requiring irAEs, the corresponding adj ORs were 3.790 (95% CI: 1.140–12.609, p = 0.030) in the LSI high group and 4.195 (95% CI: 1.123–15.673, p = 0.033) for the CT genotype. In addition, nivolumab–ipilimumab combination therapy was found to be a significant risk factor for both steroid‐requiring irAEs at any time (OR 2.932, 95% CI: 1.191–7.219, p = 0.019) and early steroid‐requiring irAEs (OR 8.500, 95% CI: 2.585–27.949, p < 0.001).

## 4 Discussion

In this single-center retrospective study, the MAF of rs16906115 was 8.3%. After adjusting for age, sex and ICI type, the presence of the rs16906115 minor allele was identified as a risk factor for all-grade irAEs. Subgroup analyses confirmed this association across multiple irAE subtypes, with no notable outliers. While LSI was not associated with all irAEs, it emerged as a significant risk factor in the steroid-treated and early steroid-treated irAE subgroups.

Groha et al. conducted a multi-cancer study among 1751 patients, demonstrating that the rs16906115 minor allele is a risk factor for all-grade irAEs, maintaining nominal significance across various irAE subtypes (13). However, in a melanoma-specific replication study by Taylor et al., an association with this allele was only reported for high-grade steroid-requiring irAEs, and its potential risk in other subgroups remains unknown. Moreover, while this association remained significant among patients receiving anti-PD-1 monotherapy, the risk increase was less pronounced in those receiving nivolumab–ipilimumab combination (14). In our study, minor allele carriage similarly conferred an increased risk of all-grade irAEs. Significant associations were also observed in the endocrine, non-cutaneous, low-grade, early-onset, and early steroid-requiring irAE subgroups. We identified minor allele carriage as a risk factor for multiple irAEs, suggesting that this SNP may not be related to a specific irAE subtype but may participate in a broader spectrum of irAE pathogenesis. Notably, our subgroup analysis diverged from that of Taylor et al. by indicating that, in minor allele carriers, nivolumab–ipilimumab combination therapy resulted in a numerically greater risk increase compared to anti-PD-1 monotherapy (14). However, this observation is based on a relatively small subgroup and therefore should be interpreted cautiously and regarded as exploratory, requiring validation in larger studies.

IL-7 plays a critical role in lymphocyte homeostasis (17). Previous research has linked IL-7R polymorphisms to autoimmune diseases such as inflammatory bowel disease, systemic lupus erythematosus, and multiple sclerosis (18). In Taylor et al.’s study, patients with melanoma who harbored the rs16906115 minor allele exhibited increased IL-7 expression in B-cells but not in other immune cell populations. Enhanced IL-7 expression has been shown to promote B- and T-cell maturation, increase effector memory T-cells, augment T-cell clonality, and improve lymphocyte stability. When IL-7 expression was controlled for, the SNP’s effect was eliminated, suggesting that its primary impact is driven by amplified IL-7 in B-cells. Taylor et al. also observed that among the minor allele carriers receiving anti-PD-1 therapy, the lymphocyte stability was higher than in the reference allele carriers (14). Consistent with these findings, our study showed no significant changes in the pre-ICI versus post-ICI lymphocyte counts in the minor allele carriers receiving anti-PD-1 monotherapy, whereas the reference allele carriers exhibited a significant decrease. Similarly, in line with Taylor et al., no notable lymphocyte decrease was detected in the patients receiving nivolumab–ipilimumab combination regardless of their allele status, possibly reflecting the pronounced CD8+ T-cell expansion induced by the dual ICI treatment (19). Additionally, the baseline lymphocyte comparisons suggested that the genetic variant does not influence the pre-ICI lymphocyte counts, implying that its impact on the lymphocyte levels becomes most evident in the context of ICI therapy (12). However, these genotype-related patterns across treatment groups were derived from subgroup comparisons and should be interpreted cautiously, particularly given the limited sample size and absence of functional evaluation of downstream pathway activation and lymphocyte dynamics. Nonetheless, previous analyses have shown that rs16906115 enhances IL-7 expression, activates the IL-7/STAT5 pathway, and modulates lymphocyte behavior, supporting the biological plausibility of our clinical findings (14).

We also evaluated the predictive value of the LSI. While the LSI did not serve as a risk factor for all irAEs, the subgroup analyses identified it as a significant risk factor for early steroid-requiring toxicities. This finding contrasts with the findings of Groha et al., who reported an association between the LSI and all irAEs (13). In our study, although the LSI showed potential in identifying patients at risk for early steroid-requiring irAEs, its discriminatory ability was moderate (AUC = 0.677), indicating that further prospective validation is necessary before clinical application. Separately, Watson et al. reported that LSI serves as a prognostic biomarker for OS in patients with solid tumors treated with ICIs. They also proposed that LSI may function as a simple and accessible peripheral blood marker to support risk stratification across solid tumors (20). It is possible that lymphocyte stability results from multiple immunogenic processes beyond the scope of IL-7 alone in patients treated with ICIs, highlighting the need for further studies to elucidate these mechanisms (12).

Given the well-established role of IL-7 in autoimmune processes, the utility of anti-IL-7R therapy has been explored in various autoimmune diseases, yielding promising preclinical study results (21, 22). However, its potential in treating irAEs remains unexamined. Future investigations should consider whether IL-7R blockade could serve as a viable therapeutic approach in clinical settings where IL-7 expression is elevated. Recent studies also suggested that pre-emptive tocilizumab administered alongside ICI therapy in patients with melanoma may reduce irAE incidence without compromising efficacy. Determining whether this strategy might be beneficial, particularly for patients at high risk of irAEs, is an important direction for future research (23).

Identifying high-risk patients through carriage of the rs16906115 minor allele could prove invaluable for the management of melanoma patients, especially in neoadjuvant and adjuvant contexts where surgical cure is achievable. Neoadjuvant immunotherapy is becoming integral to the treatment paradigm for stage III melanoma, offering improved pathological responses and potentially enhanced long-term outcomes. In this regard, both combination therapy (Nivolumab plus Ipilimumab) and monotherapy (pembrolizumab) are viable neoadjuvant options (24, 25); however, no studies have conclusively demonstrated the clear superiority of one approach over the other. In patients who are deemed to face a high risk of irAEs, pembrolizumab monotherapy could thus be prioritized to mitigate toxicity. Furthermore, as there is no definitive evidence that ICI therapy results in superior overall survival than anti-BRAF therapy in the adjuvant treatment of BRAF-positive melanoma, clinicians might consider anti-BRAF treatments for individuals with a high risk of irAEs (26). In the metastatic setting, while combination ICI therapy confers substantial survival benefits, it may be prudent to choose anti-PD-1 monotherapy over combination therapy for patients who have a higher risk of severe irAEs (27). Nonetheless, these clinical considerations remain speculative and require validation through prospective trials before being incorporated into routine clinical practice.

Our study is limited by its retrospective design, which may have led to the underestimation of low-grade toxicities. Although the overall sample size was sufficient to detect statistically significant differences in the entire cohort, subset analyses focusing on specific treatments and irAE subtypes may have been underpowered, potentially obscuring certain associations. The absence of TT genotype and the small number of CT carriers (n=16) in our study cohort limit the interpretation of subgroup analyses and comparisons based on treatment type or genotype. However, as the TT genotype frequency in European ancestry is ~0.7%, this distribution is statistically expected and consistent with population-level data (28). Consequently, larger prospective cohort studies are needed to clarify the relationships among rs16906115, lymphocyte stability, and the irAE risk across different ethnic groups.

## 5 Conclusion

Our findings corroborate previous European data by demonstrating that both carriage of the rs16906115 minor allele and lymphocyte stability are associated with an increased risk of irAEs, now validated in a distinct ethnic cohort. In our melanoma-specific population, this SNP conferred not only a statistically significant risk of all-grade irAEs but also a distinct elevation of the risk in terms of various irAE subtypes. Additionally, our data extend prior findings by indicating that the minor allele may also increase the irAE risk in the context of combination ICI therapy, not solely anti-PD-1 monotherapy. Hence, IL-7 SNPs exhibit potential for identifying patients at high risk of irAE and guiding therapeutic decision-making in melanoma cases. However, further validation in larger multi-ethnic cohorts is necessary before this biomarker can be adopted in routine clinical practice.

## Data Availability

The datasets presented in this study can be found in online repositories. The names of the repository/repositories and accession number(s) can be found below: https://doi.org/10.5281/zenodo.15261524, 15261524.

## Supplementary material

Supplementary Table 1 Logistic Regression Analysis of Factors Associated with irAE subtypes

Supplementary Figure 1 ROC curve for Lymphocyte stability index
