# Supplementary material for: Predicting immune-related adverse events in patients with melanoma: the role of interleukin-7 rs16906115 polymorphism and lymphocyte dynamics
Source: Front Immunol. 2025 Jun 26;16:1616325. doi: 10.3389/fimmu.2025.1616325 (PMC12240767; doi:10.3389/fimmu.2025.1616325)
Supplement: Supplementary Table 1 — Logistic Regression Analysis of Factors Associated with irAE subtypes [file Table1.docx]

Supplementary Material

## Supplementary Tables

**Supplementary Table S1.** Logistic Regression Analysis of Factors Associated with irAE subtypes

| **Table S1. Logistic Regression Analysis of Factors Associated with irAE subtypes** | | | | |
| --- | --- | --- | --- | --- |
|  | Steroid-requiring irAE | | Early steroid-requiring irAE | |
|  | OR (95 % CI) | *p* | OR (95 % CI) | *p* |
| Age, years | 1.002 (0.970-1.04) | 0.900 | 0.998 (0.962-1.03) | 0.896 |
| Gender (male vs female) | 1.131 (0.454-2.815) | 0.791 | 1.191 (0.429-3.307) | 0.737 |
| Histological subtype  (acral vs nonacral) | 1.817 (0.380-8.693) | 0.454 | 2.958 (0.607-14.417) | 0.180 |
| BRAF status (wild vs mutant) | 1.240 (0.488-3.149) | 0.651 | 1.061 (0.381-2.956) | 0.909 |
| Brain metastasis (yes vs no) | 1.173 (0.272-5.051) | 0.830 | 1.917 (0.436-8.418) | 0.389 |
| Liver metastasis (no vs yes) | 2.652 (0.549-12.811) | 0.225 | 1.633 (0.333-8.017) | 0.546 |
| Bone metastasis (yes vs no) | 1.746 (0.626-4.872) | 0.287 | 2.385 (0.805-7.068) | 0.117 |
| ICI type (nivo-ipi vs anti pd-1) | 2.932 (1.191-7.219) | 0.019 | 8.500 (2.585-27.949) | < 0.001 |
| LDH (>ULN vs normal) | 1.119 (0.394-3.176) | 0.833 | 1.543 (0.506-4.702) | 0.446 |
| Albumin (low vs high) | 1.353 (0.488-3.756) | 0.561 | 2.476 (0.848-7.231) | 0.097 |
| CRP (high vs low) | 1.481 (0.502-4.371) | 0.477 | 1.909 (0.599-6.081) | 0.274 |
| Lymphocyte stability index (high vs low) | 3.635 (1.453-9.092) | 0.006 | 6.031 (1.986-18.317) | 0.002 |
| IL-7 rs16906115 (CT vs CC) | 2.810 (0.936-8.434) | 0.066 | 3.667 (1.168-11.508) | 0.026 |

**Supplementary Figures**

**Supplementary Figure S1.** ROC curve for Lymphocyte stability index
