# Supplementary figures and images for: Predicting immune-related adverse events in patients with melanoma: the role of interleukin-7 rs16906115 polymorphism and lymphocyte dynamics
Source: Front Immunol. 2025 Jun 26;16:1616325. doi: 10.3389/fimmu.2025.1616325 (PMC12240767; doi:10.3389/fimmu.2025.1616325)

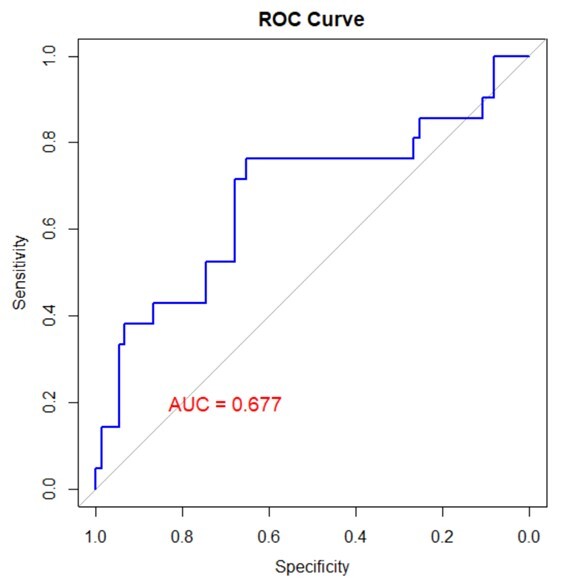

Supplement: Supplementary Figure 1 — ROC curve for Lymphocyte stability index [file Image1.jpeg]
